# Modeling the Life Cycle of the Intramitochondrial Bacterium “*Candidatus* Midichloria mitochondrii” Using Electron Microscopy Data

**DOI:** 10.1128/mBio.00574-21

**Published:** 2021-06-22

**Authors:** Francesco Comandatore, Giacomo Radaelli, Sebastiano Montante, Luciano Sacchi, Emanuela Clementi, Sara Epis, Alessandra Cafiso, Valentina Serra, Massimo Pajoro, Domenico Di Carlo, Anna Maria Floriano, Fabrizia Stavru, Claudio Bandi, Davide Sassera

**Affiliations:** a Romeo ed Enrica Invernizzi Pediatric Research Center, Department of Biomedical and Clinical Sciences Luigi Sacco, Università di Milano, Milan, Italy; b Dipartimento di Biologia e Biotecnologie L. Spallanzani, Università degli Studi di Pavia, Pavia, Italy; c Romeo ed Enrica Invernizzi Pediatric Research Center, Department of Biosciences, Università di Milano, Milan, Italy; d Department of Veterinary Medicine, Università di Milano, Milan, Italy; e Unité des Interactions Bactéries-Cellules, Institut Pasteur, Paris, France; University of Sydney; Korea Advanced Institute of Science and Technology

**Keywords:** “*Candidatus* Midichloria mitochondrii, *Ixodes ricinus*, mitochondrial network, *Bdellovibrio*-like hypothesis, endosymbiosis

## Abstract

“*Candidatus* Midichloria mitochondrii” is a Gram-negative bacterium that lives in strict intracellular symbiosis with the hard tick Ixodes ricinus, forming one of the most intriguing endosymbiosis described to date. The bacterium is capable of durably colonizing the host mitochondria, a peculiar tropism that makes “*Ca*. Midichloria mitochondrii” a very interesting tool to study the physiology of these cellular organelles. The interaction between the symbiont and the organelle has, however, been difficult to characterize. A parallelism with the predatory bacterium Bdellovibrio bacteriovorus has been drawn, suggesting the hypothesis that “*Ca*. Midichloria mitochondrii” could prey on mitochondria and consume them to multiply. We studied the life cycle of the bacterium within the host oocytes using a multidisciplinary approach, including electron microscopy, molecular biology, statistics, and systems biology. Our results were not coherent with a predatory-like behavior by “*Ca*. Midichloria mitochondrii” leading us to propose a novel hypothesis for its life cycle. Based on our results, we here present a novel model called the “mitochondrion-to-mitochondrion hypothesis.” Under this model, the bacterium would be able to move from mitochondrion to mitochondrion, possibly within a mitochondrial network. We show that this model presents a good fit with quantitative electron microscopy data.

## INTRODUCTION

A number of bacterial endosymbionts interacting with a wide range of hosts have been described. Among them, one of the most intriguing is “*Candidatus* Midichloria mitochondrii,” capable of thriving within host mitochondria. “*Ca*. Midichloria mitochondrii” is a Gram-negative alphaproteobacterium and a member of the order *Rickettsiales*. “*Ca*. Midichloria mitochondrii” is the most abundant member of the microbiome of the hard tick Ixodes ricinus (1), an important arthropod vector of infections in Europe, including Lyme disease (2, 3). The bacterium is present with ∼100% prevalence in adult females and ∼40% prevalence in adult males (4), and real-time PCR experiments showed that “*Ca*. Midichloria mitochondrii” is present throughout the tick life cycle (eggs, larvae, nymphs, and adults) (5). In particular, in adult females, most of the “*Ca*. Midichloria mitochondrii” population is localized in the ovaries, while a small portion is found in other organs (6, 7). The ∼100% prevalence of “*Ca*. Midichloria mitochondrii” in female ticks throughout their geographic distribution is suggestive of a mutualistic relationship (4), but the role of “*Ca*. Midichloria mitochondrii” in the *I. ricinus* biology is still not clear. Analysis of the bacterial genome revealed that “*Ca*. Midichloria mitochondrii” possesses the genes for the biosynthesis of B vitamins and for a *cbb*_3_-type cytochrome *c* oxidase variant which, having high affinity for oxygen, allows microbial respiration under microaerobic conditions (8). This suggests that the bacterium could be an important source of vitamins for the tick and possibly also an ATP source under low-oxygen conditions (8).

Electron microscopy observations of tick oocytes showed symbiotic bacteria both free in the cytoplasm and inside the intermembrane space of mitochondria, with cases of organelles colonized by up to a dozen bacteria (9). Mitochondria containing multiple symbionts appeared to be degraded and/or swollen, with the matrix only occupying a small portion of the entire organelle. These observations led Sacchi and colleagues (9) to propose that “*Ca*. Midichloria mitochondrii” could have a behavior similar to that of predatory bacterium (“*Bdellovibrio*-like” model). These are free-living bacteria that invade other prokaryotes, localized between the cell wall and the membrane (i.e., the periplasm, which is topologically equivalent to the mitochondrial intermembrane space). After invasion, *Bdellovibrio* consumes their prey bacterium, multiplies therein, and finally bursts out in high numbers (10). In the *Bdellovibrio*-like model, “*Ca*. Midichloria mitochondrii” bacteria localized in the cytoplasm would invade noncolonized mitochondria, replicate within them, and then return to the cytoplasm after mitochondrial lysis (9). This hypothesis is also based on the consideration that mitochondria evolved from Gram-negative bacteria and still maintain common features with them, including a double membrane (11).

At the state of the art, *in vitro* culture of “*Ca*. Midichloria mitochondrii” in *I. ricinus* oocytes is not available; thus, it is impossible to directly observe the “*Ca*. Midichloria mitochondrii” life cycle. On the other hand, the life cycle of predatory bacteria has been deeply investigated, both experimentally and by mathematical models (12). The different mathematical models present in the literature are concordant in describing an oscillatory equilibrium for most of the prey-predatory systems, including that of *Bdellovibrio* (12). Additionally, in nutrient-rich environments, the amplitude of the oscillations is expected to be bigger than when nutrients are scarce (12). Starting from this information, we decided to test the *Bdellovibrio*-like hypothesis using quantitative electron microscopy data collected from *I. ricinus* oocytes.

Our results indicate that “*Ca*. Midichloria mitochondrii” probably does not have a *Bdellovibrio*-like life cycle. We thus considered another possible model, in which the bacterium would move from mitochondrion to mitochondrion. The reasoning behind this hypothesis is that mitochondria are often interconnected with each other, forming a dynamic network (13, 14) instead of being discrete units. The existence of a proper mitochondrial network has never been tested in ticks, but this phenomenon is widespread in a number of organisms, including arthropods (15). The model of “*Ca*. Midichloria mitochondrii” movement from one mitochondrion to another, developed here, was tested mathematically through simulations and resulted to fit our experimental data.

## RESULTS

### Electron microscopy and molecular biology.

**FIG 1:**
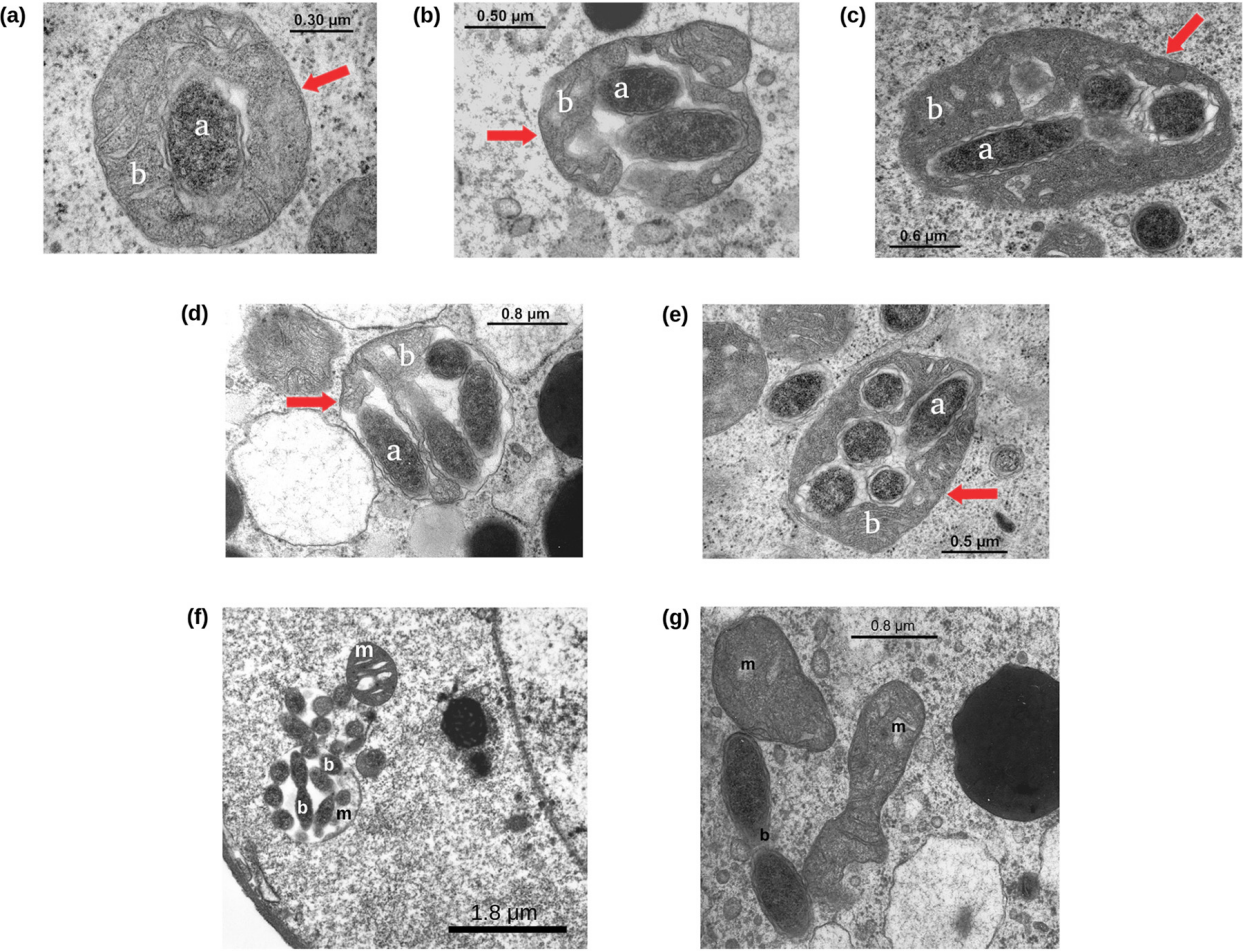
Transmission electron microscopy observation of “*Ca*. Midichloria mitochondrii” bacteria in Ixodes ricinus oocytes. TEM images of mitochondria of Ixodes ricinus oocytes colonized by at least one (a), two (b), three (c), four (d), and five (e) “*Ca*. Midichloria mitochondrii” cells. In each photo, the red arrow indicates the mitochondrial membrane, the letter “a” indicates an intramitochondrial *M. mitochondrii* cell, and the letter “b” indicates the mitochondrial matrix. “*Ca*. Midichloria mitochondrii” cells possibly in replication within a mitochondrion (f) and in the cytoplasm (g); b, bacterium; m, mitochondrion.

A total of 11 ticks were prepared for transmission electron microscopy (TEM), and a total of 71 oocyte sections were observed, 42 at previtellogenic (P) and 29 at late-previtellogenic (LP) development stages (for details about the oocytes, see Table S1 available at https://github.com/FrancescoComandatore/M.mitochondrii_TEM_count/blob/main/TableS1.xls; for details about the sampled ticks, see Table S2 at https://github.com/FrancescoComandatore/M.mitochondrii_TEM_count/blob/main/TableS2.xls). The number of “*Ca*. Midichloria mitochondrii” outside mitochondria (cytoplasmic “*Ca*. Midichloria mitochondrii”) and the number of colonized mitochondria were counted, for a total of 12,068 mitochondria and 7,805 “*Ca*. Midichloria mitochondrii” units (see Table S1 at https://github.com/FrancescoComandatore/M.mitochondrii_TEM_count/blob/main/TableS1.xls). Colonized mitochondria were differentiated based on colonization level (mitochondrion colonization level [MCL], i.e., the number of mitochondria containing zero to five or more bacteria) (Fig. 1a to e). These extensive TEM observations showed a very limited number of “*Ca*. Midichloria mitochondrii” bacteria possibly in replication, although the possibility of these being just pairs of bacteria in close proximity cannot be ruled out. Specifically, two possible replication events within a mitochondrion and nine replications outside mitochondria were detected (Fig. 1f and g). All observations and counts were obtained from oocyte sections; therefore, the number of counted mitochondria and bacteria likely represent an underestimation of the real numbers per cell. This is an inherent limit of the method used. Unfortunately, at the state of the art, the spatial distribution of mitochondria and “*Ca*. Midichloria mitochondrii” cells in the *I. ricinus* oocytes is unknown. Thus, without *a priori* information and for parsimony, we can only consider mitochondria and “*Ca*. Midichloria mitochondrii” cells as homogeneously distributed in the oocyte cytoplasm. Under these assumptions, the ratios between mitochondria and “*Ca.* Midichloria mitochondrii” cells should be only slightly affected by section sampling. Thus, we used ratios, and not the absolute numbers, in all the analysis described below. From here on, we will refer to oocyte sections as oocytes for sake of simplicity.

Two semiengorged ticks were dissected for molecular biology analysis, and from each one, five groups of ∼10 oocytes at the previtellogenic development stage were retrieved. The real-time PCR quantifications of *COII* (mitochondrial cytochrome oxidase II of *I. ricinus*, harbored by the mitochondrial DNA) and *cal* (calreticulin gene of *I. ricinus*, present in the nuclear genome) genes for each group of oocytes are reported in Table S3 at https://github.com/FrancescoComandatore/M.mitochondrii_TEM_count/blob/main/TableS3.xls. The median *COII*/*cal* value was 1,285.6, corresponding to an estimation of mitochondria per cell. This number is roughly an order of magnitude higher than the mitochondrial counts obtained by TEM. We explain this discrepancy considering that the TEM procedure only counts the mitochondria present in a section of the entire oocyte. Additional PCR experiments showed the absence of *Coxiella* spp./*Coxiella*-like and *Rickettsia* spp./*Rickettsia*-like bacteria in these samples (see Fig. S1 in the supplemental material).

10.1128/mBio.00574-21.1 FIG S1 PCR gel for *Coxiella* spp./*Coxiella*-like and *Rickettsia* spp./*Rickettsia*-like. Agarose gel electrophoresis (2% agarose) of PCR-amplified products using species-specific PCR primers for *Coxiella* spp./*Coxiella*-like and *Rickettsia* spp./*Rickettsia*-like bacteria. Samples from 1 to 10 are sorted as in Table S4, available at https://github.com/FrancescoComandatore/M.mitochondrii_TEM_count/blob/main/TableS4.xls. C+, positive control; neg, negative control. Download FIG S1, TIF file, 0.2 MB. Copyright © 2021 Comandatore et al. 2021 Comandatore et al. https://creativecommons.org/licenses/by/4.0/ This content is distributed under the terms of the Creative Commons Attribution 4.0 International license.

### Comparison of oocytes at different developmental stages.

The total numbers of “*Ca*. Midichloria mitochondrii” cells were significantly different between oocytes at the two stages (LP and P) (Wilcoxon test, *P* value < 0.05; medians, 77 in P and 90 in LP oocytes). A similar trend was found for both the total numbers of intramitochondrial bacteria (Wilcoxon test, *P* value < 0.05; P median, 11.5; LP median, 22) and the frequency of intramitochondrial bacteria (Wilcoxon test, *P* value < 0.05; P median, 0.11; LP median, 0.21).

Similarly, the total numbers of mitochondria significantly differed between P and LP stages (Wilcoxon test, *P* value < 0.05; P median, 110.5; LP median, 179) as did the total numbers of colonized mitochondria (Wilcoxon test, *P* value < 0.05; P median, 8; LP median, 22). On the other hand, the frequency of colonized mitochondria did not vary significantly between the developmental stages (Wilcoxon test, *P* value > 0.05; P median, 0.074; LP median, 0.085).

Finally, the frequency of mitochondria colonized by at least one, two, three, four, or five “*Ca*. Midichloria mitochondrii” bacterial cells did not change significantly between P and LP stages (Wilcoxon test, *P* value > 0.05) (see Fig. S2).

10.1128/mBio.00574-21.2 FIG S2 Boxplots of the frequencies of the different mitochondrial colonization levels in oocytes at previtellogenic (green) and late-previtellogenic (blue) developmental stages. The complementary cumulative distributions (CCDs) of the mitochondrial colonization levels of oocytes are shown. Download FIG S2, TIF file, 0.4 MB. Copyright © 2021 Comandatore et al. 2021 Comandatore et al. https://creativecommons.org/licenses/by/4.0/ This content is distributed under the terms of the Creative Commons Attribution 4.0 International license.

### Regression analysis of the frequencies of cytoplasmic and intramitochondrial “*Ca*. Midichloria mitochondrii” and of free and colonized mitochondria.

Regression analysis showed that the numbers of cytoplasmic “*Ca*. Midichloria mitochondrii” and noncolonized mitochondria were linearly correlated in oocytes at both previtellogenic and late-previtellogenic stages (linear regression, *P* value < 0.0001 for both stages) (see Fig. S3). The number of observed colonized mitochondria was lower than the number of “*Ca*. Midichloria mitochondrii” and noncolonized mitochondria in all the observed oocytes but one (see Fig. S4).

10.1128/mBio.00574-21.3 FIG S3 Correlation between frequency of colonized mitochondria and frequency of intramitochondrial “*Ca*. Midichloria mitochondrii.” The frequency of colonized mitochondrial and intramitochondrial bacteria were calculated on the basis of the data collected by TEM experiments for each oocyte. The regression analysis shows significant correlations (linear regression, *P* value < 0.05) for oocytes at both previtellogenic (points and regression line in green) and late-previtellogenic (blue) developmental stages and considering all the points together (regression line in black). Download FIG S3, TIF file, 0.3 MB. Copyright © 2021 Comandatore et al. 2021 Comandatore et al. https://creativecommons.org/licenses/by/4.0/ This content is distributed under the terms of the Creative Commons Attribution 4.0 International license.

10.1128/mBio.00574-21.4 FIG S4 Numbers of cytoplasmic “*Ca*. Midichloria mitochondrii” cells (red), noncolonized mitochondria (blue), and colonized mitochondria (in green) relative to the oocytes at previtellogenic developmental stage (left) and at the late-previtellogenic stage (right). In each of the graphs, the oocytes are ordered as the relative sum of counted mitochondria and bacteria. Download FIG S4, TIF file, 0.5 MB. Copyright © 2021 Comandatore et al. 2021 Comandatore et al. https://creativecommons.org/licenses/by/4.0/ This content is distributed under the terms of the Creative Commons Attribution 4.0 International license.

We further detected that the frequency of colonized mitochondria was positively correlated with the frequency of intramitochondrial “*Ca*. Midichloria mitochondrii” in oocytes at both development stages (see Fig. S5) (linear regression, *P* value < 0.05 for both).

10.1128/mBio.00574-21.5 FIG S5 Correlation between number of noncolonized mitochondria and the number of cytoplasmic “*Ca*. Midichloria mitochondrii” cells. The regression analyses show significant correlations (linear regression, *P* value < 0.05) for oocytes at both previtellogenic (points and regression line in blue) and late-previtellogenic (green) developmental stages. Download FIG S5, TIF file, 0.2 MB. Copyright © 2021 Comandatore et al. 2021 Comandatore et al. https://creativecommons.org/licenses/by/4.0/ This content is distributed under the terms of the Creative Commons Attribution 4.0 International license.

### Linear regression of prey and predator amounts in an oscillatory equilibrium according to the Lotka-Volterra model.

Sacchi and colleagues (9) proposed that “*Ca*. Midichloria mitochondrii” could invade and lyse mitochondria with a behavior comparable to that of predatory bacteria (*Bdellovibrio*-like model). Here, we evaluated the probability of obtaining a significant linear correlation between the numbers of prey (mitochondria) and predators (cytoplasmic “*Ca*. Midichloria mitochondrii”) in a prey-predator system in oscillatory equilibrium. We studied an oscillatory equilibrium of the Lotka-Volterra model using a bootstrap approach with 10,000 replicates, considering different numbers of sampling points (from 10 to 150). We found that the frequency of bootstrap replicates with significant linear regression decreases as the number of sample time points increases (see Fig. S6). The frequency goes below 0.01 for ≥60 time points.

10.1128/mBio.00574-21.6 FIG S6 Frequency of bootstrapping resamples with prey and predators linearly significantly correlated. An oscillatory equilibrium of the Lotka-Volterra model was studied to evaluate how the number of sampling points affects the probability of obtaining a significant prey-predator linear regression. N random time points of the oscillatory system were collected, and the linear regression *P* value was computed; 10,000 bootstrap replicates of this analysis were computed for N equal to 10, 20, 30, 40, 50, 60, 70, 80, 90, 100, 110, 120, 130, 140, and 150. In the graph, the frequency of the 10,000 bootstrap replicates returning significant correlation (using 0.05, 0.01, and 0.001 *P* value thresholds) against the number of the sampled time points is shown. Starting from the top, the first horizontal dashed line corresponds to the frequency of 0.05 and the second to 0.01. The vertical dashed line corresponds to 71, the number of oocytes included in the study. Download FIG S6, TIF file, 0.3 MB. Copyright © 2021 Comandatore et al. 2021 Comandatore et al. https://creativecommons.org/licenses/by/4.0/ This content is distributed under the terms of the Creative Commons Attribution 4.0 International license.

### Power law distribution of the mitochondrial colonization level.

Forty of the 71 analyzed oocytes displayed at least three mitochondrial colonization levels (MCLs); thus, the relative complementary cumulative distributions (CCDs) of the frequency of MCL (i.e., frequency of mitochondria colonized by 0, 1, 2, 3, 4, 5, or more “*Ca*. Midichloria mitochondrii” cells) were tested to see if they fit a power law. All of them showed a power law trend (see Table S4 for details, available at https://github.com/FrancescoComandatore/M.mitochondrii_TEM_count/blob/main/TableS4.xls). Furthermore, the homoscedasticity of the residuals for the CCD of each oocyte was verified for each sample (Breusch-Pagan test, *P* value > 0.05) as well as the normality of the residuals (Shapiro test, *P* value > 0.05). The nonautocorrelation of the residuals was tested using the Ljung-Box test, resulting in a *P* value of >0.05 for all samples and thus verifying the independent distribution of the residuals.

Once the validity of the fit was confirmed, the alpha parameters estimated for each CCD were compared between P and LP oocytes through both Wilcoxon and *t* tests. The Wilcoxon test returned a *P* value of >0.05, showing the absence of a significant difference between the two populations. Before performing the *t* test, the normality of the P and LP populations was verified using the Shapiro test (resulting in a *P* value of >0.05), and variance uniformity was verified using the Bartlett test (resulting in a *P* value of >0.05). Then, the *t* test resulted in a *P* value of >0.05, confirming that there is no significant difference between alpha calculated for P and LP oocytes.

**FIG 2:**
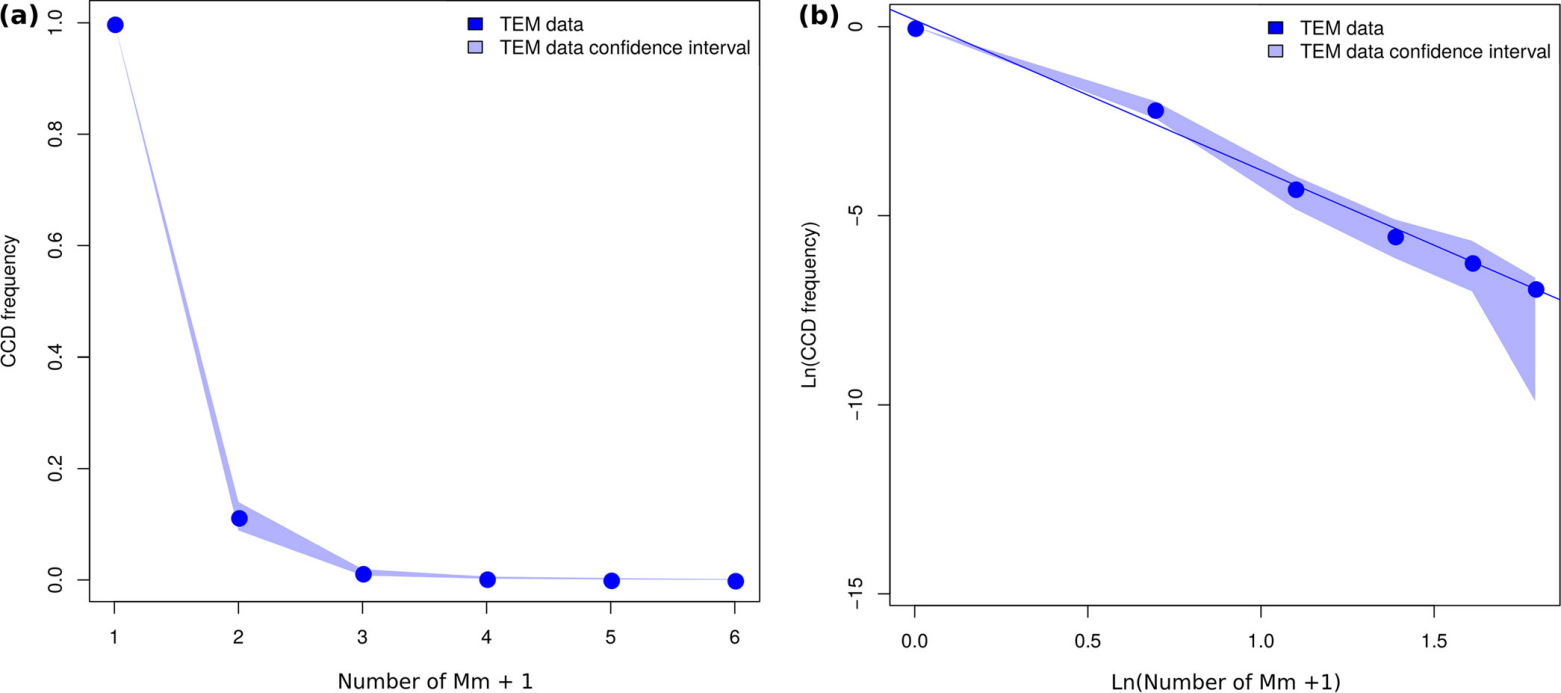
Complementary cumulative distributions of the different levels of mitochondrial colonization. (a) Complementary cumulative distribution (CCD) of the mitochondrial colonization levels; *x* axis shows the number of intramitochondrial “*Ca*. Midichloria mitochondrii” (Mm) plus 1; *y* axis shows the frequency of mitochondria colonized by at least Mm plus 1 bacteria. (b) Log-log plot of the CCD. The Mm plus 1 scale (i.e., 1 for bacterium-free mitochondria, 2 for mitochondria colonized by one bacterium, and so on) has been used to allow the transformation of the CCD plot to the log-log plot. In both graphs, the line connecting the CCD points is colored blue, while the CCD confidence interval is reported in light blue. This graph shows that the distribution of the frequency of mitochondrial colonization levels follows a power law.

The average CCD among all CCDs obtained by the data set was calculated as previously described and resulted in a power law distribution (see Fig. 2) with an *R*^2^ of 0.993 and a *P* value of <0.001, for an alpha parameter value of 3.97.

### Mathematical simulation of the spread of “*Ca*. Midichloria mitochondrii” through the mitochondrial network.

**FIG 3:**
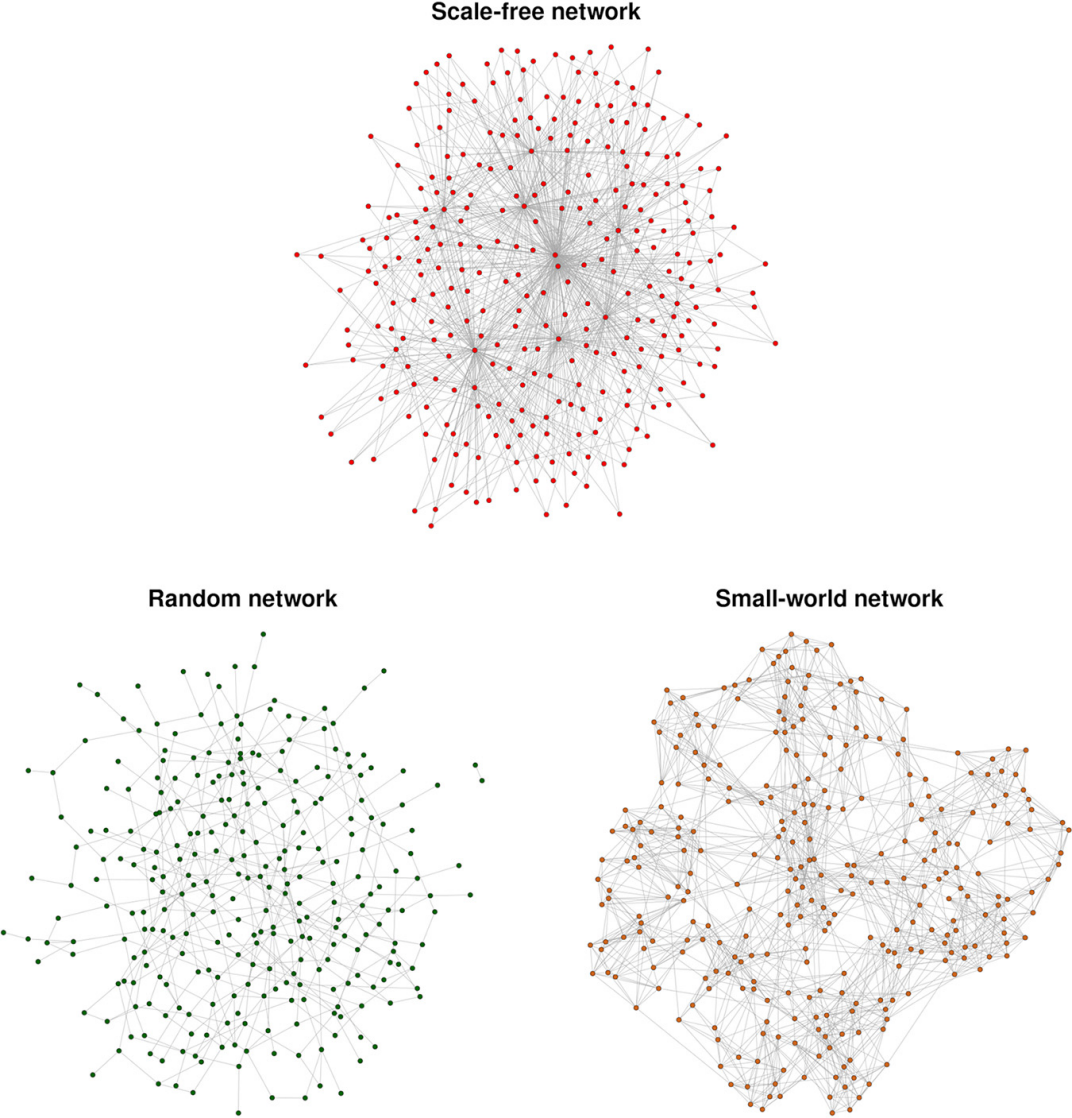
Scale-free, random, and small-world graphs characterized by different topologies.

The hypothesis that “*Ca*. Midichloria mitochondrii” can move from one mitochondrion to another was tested using a stochastic simulation approach. For the sake of simplicity, mitochondria were considered to be connected in a static network. Three different topologies were considered: scale free, small world, and random (Fig. 3). The random movement of the bacterium in the network was simulated for 1,000 steps and 10,000 repetitions for each of the topologies. At each step, the frequency CCD of the MCL was calculated.

Considering that the average number of mitochondria per oocyte estimated by real-time PCR assays (see above) was 1,285.6, we set the number of nodes at 1,000, and the resulting K parameter value was 160 (see Materials and Methods).

At the end of each of the simulations, the average CCD of each MCL was at equilibrium (see Fig. S7); thus, the average CCD calculated at the last step (step 1,000) was considered representative of the system. For each of the four simulations, the obtained average CCD followed a power law trend (log-log linear regression, *P* value < 0.05), significantly fitting the average CCD calculated from TEM experiments data (Spearman’s rank test, *P* value < 0.05).

10.1128/mBio.00574-21.7 FIG S7 Average frequencies of mitochondria colonized by “*Ca*. Midichloria mitochondrii” cells calculated in each step of the mitochondrion-to-mitochondrion hypothesis simulations. (a, b, c, d, and e) Average frequencies of mitochondria colonized by at least one, two, three, four, or five “*Ca*. Midichloria mitochondrii” cells in each of the 1,000 steps of the 10,000 independent simulations, respectively. In each simulation, bacteria are randomly moved from a node of the mitochondrial network to a neighbor node with a probability of 0.1. The average frequencies are reported with a solid line, and the relative maximum and minimum values are reported with dashed lines, all of them colored on the basis of the topology of the network used in the simulation. Values relative to simulations performed on scale-free mitochondrial networks are red, those for random networks are green, and those for small-world networks are brown. The average frequency of mitochondria colonized by at least two “*Ca*. Midichloria mitochondrii” cells calculated from TEM data is reported with a black solid line. Download FIG S7, TIF file, 1.4 MB. Copyright © 2021 Comandatore et al. 2021 Comandatore et al. https://creativecommons.org/licenses/by/4.0/ This content is distributed under the terms of the Creative Commons Attribution 4.0 International license.

**FIG 4:**
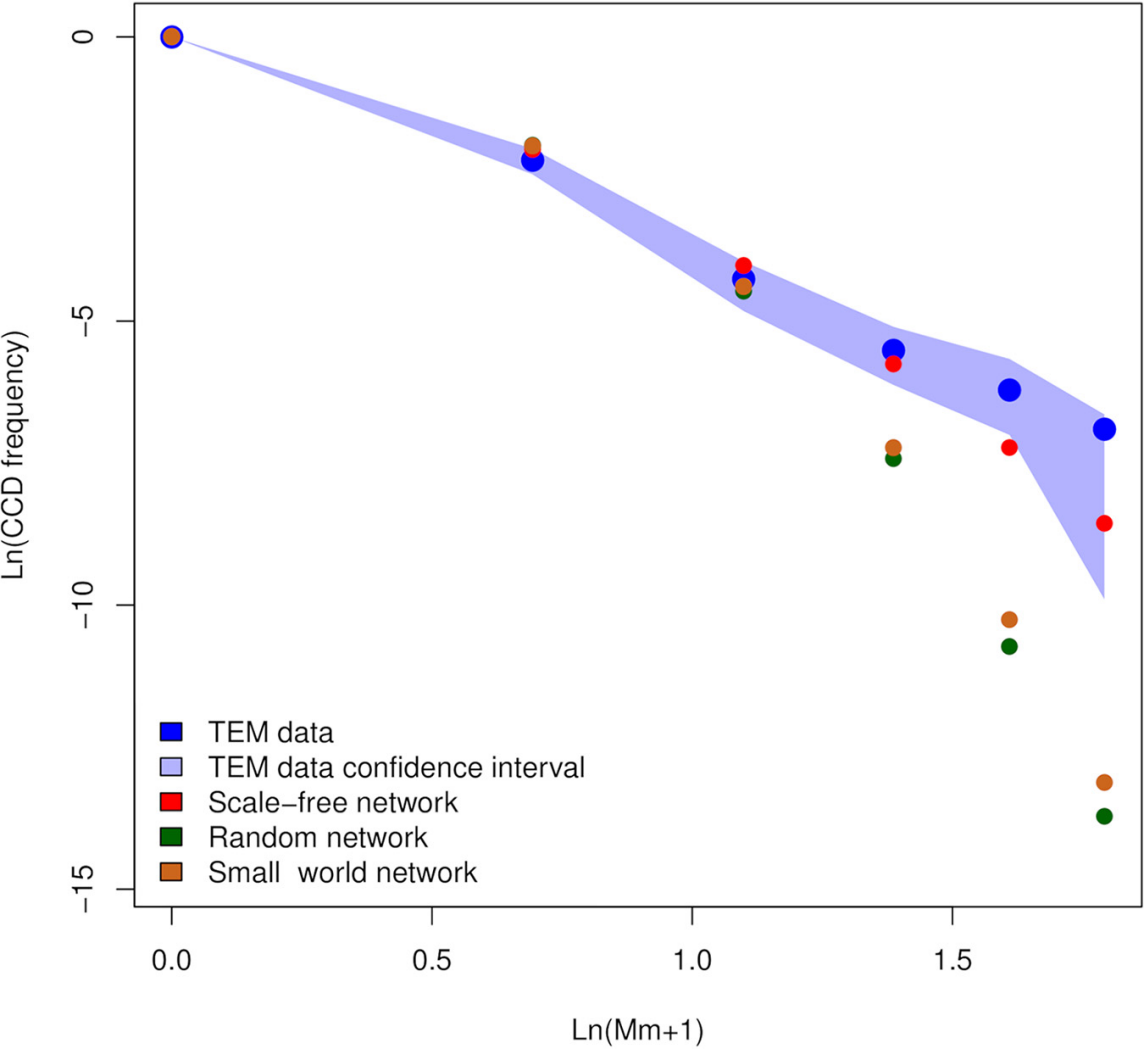
Complementary cumulative distribution of the different levels of mitochondrial colonization obtained from TEM data and from simulations. The complementary cumulative distribution (CCD) of the mitochondrial colonization levels (i.e., the frequency of mitochondria colonized by at least Mm plus 1 bacteria) obtained from TEM data is reported in blue, and the relative confidence interval is in light blue. The CCDs calculated from the simulations performed to test the mitochondrial network hypothesis on different mitochondrial network topologies are also reported, colored red for scale-free topology, green for random topology, and brown for small-world topology. All the simulations fit well the frequencies of mitochondria colonized by at least zero, one, and two bacteria; indeed, the estimated values fall within the confidence interval of the TEM data. On the other hand, only the simulations of scale-free networks fit well the frequencies of mitochondria colonized by more than two bacterial cells.

Although scale-free, small-world, and random network simulations returned results consistent with TEM data, the average CCD obtained from the scale-free simulation better fit the TEM data (see Fig. 4).

## DISCUSSION

### Rationale of the study.

“*Ca*. Midichloria mitochondrii” is an intriguing endosymbiont, being able to colonize not only the host oocytes’ cytoplasm (as many other endosymbionts do, ensuring vertical transmission) but also their mitochondria. This unique tropism probably led the bacterium to establish a unique relationship with host mitochondria, making “*Ca*. Midichloria mitochondrii” a very interesting tool to study the physiology of the organelle.

A first model for the “*Ca*. Midichloria mitochondrii” life cycle was proposed by Sacchi and colleagues (9) on the basis of qualitative electron microscopy observations. Considering the presence of “*Ca*. Midichloria mitochondrii” bacteria within both the cytoplasm and mitochondria of host cells and that single mitochondria were found to be colonized by up to 10 bacteria, they proposed that the bacterium could be able to invade mitochondria, replicate within them, lyse them, and then return to the cytoplasm (9). Such a life cycle bears similarity to that of predatory bacteria, such as Bdellovibrio bacteriovorus. Sacchi and colleagues thus called the model *Bdellovibrio*-like (9). This model has not been tested because, at the state of the art, *in vitro* cultivation of Ixodes ricinus oocytes is not available. In this study, we tested the model using quantitative data and a multidisciplinary approach, including electron microscopy, molecular biology, statistics, and systems biology.

### Intramitochondrial “*Ca*. Midichloria mitochondrii” are not detrimental to mitochondria.

Interestingly, we found that the average frequency of intramitochondrial “*Ca*. Midichloria mitochondrii” cells increases, from 0.11% to 0.21%, from the previtellogenic to the late-previtellogenic stage of oocytes. We envision two possible explanations for this result: either the bacteria invade additional mitochondria or they replicate within them. However, from all of our images, we found only two cases of “*Ca*. Midichloria mitochondrii” possibly in replication within mitochondria, favoring the first hypothesis.

Furthermore, we found that the frequency of colonized mitochondria is positively correlated with the frequency of intramitochondrial bacteria in the oocytes (see Fig. S3 in the supplemental material). This result suggests that when the frequency of intramitochondrial “*Ca*. Midichloria mitochondrii” increases, there is no direct compensatory effect in mitochondria, i.e., the number of mitochondria does not increase, which would maintain a stable frequency of colonized mitochondria. This phenomenon suggests that the population of intramitochondrial “*Ca*. Midichloria mitochondrii” could be nondetrimental to host mitochondria.

### A common mechanism underlies the “*Ca*. Midichloria mitochondrii” life cycle in tick oocytes.

The life cycle of “*Ca*. Midichloria mitochondrii” within the *I. ricinus* oocytes cannot be directly observed because of the current inability to maintain tick oocytes *in vitro*. We thus used electron microscopy to quantify symbionts and organelles in 71 sections of oocytes collected from 11 adult ticks. This sampling allows TEM observations to be considered independent and to randomly sample moments of the “*Ca*. Midichloria mitochondrii” life cycle within the oocytes. Interestingly, these observations show a conserved pattern among the oocytes, suggesting that the “*Ca*. Midichloria mitochondrii” life cycle is similar in all of the oocytes.

Although the frequency of intramitochondrial “*Ca*. Midichloria mitochondrii” cells significantly changed between P and LP oocyte stages, we found a strongly conserved pattern among the complementary cumulative distributions (CCDs) calculated for all of the oocytes. In particular, we found that all of them (or, more precisely, all of those presenting at least three levels of colonization, i.e., a CCD with at least three points) follow a power law distribution and that the relative alpha parameters did not change significantly among the oocytes at different development stages. This suggests that there is a common biological mechanism underlying the distribution of “*Ca*. Midichloria mitochondrii” cells among mitochondria in tick oocytes.

Moreover, it means that we can merge all the collected TEM data to calculate the average CCD of “*Ca*. Midichloria mitochondrii” within the oocytes in order to obtain a unique equation for the description of the frequency of the bacterium within the “average oocyte.” The equation is

(1) $$f(\text{MCL})={\text{MCL}}^{-3.97},$$

where *f*(MCL) is the expected frequency of a certain MCL status within the oocyte.

### Testing the *Bdellovibrio-*like model.

The *Bdellovibrio*-like model envisions a “predatory” behavior of “*Ca*. Midichloria mitochondrii” toward mitochondria. The bacterium would be invading them, replicating within them, and escaping from them by provoking mitochondrial lysis.

The *Bdellovibrio* life cycle represents an interesting prey-predator system, one that has been deeply investigated by mathematical modeling (12). The different mathematical models present in the literature are focused on different aspects of the system, but they are concordant in describing an oscillatory equilibrium (12). The prey-predator phase graph of such an oscillatory system is a closed line similar to a circle.

To test the *Bdellovibrio*-like model in light of our data, we consider “*Ca*. Midichloria mitochondrii” as the predator and the mitochondria as prey.

Our TEM data show that the number of cytoplasmic “*Ca*. Midichloria mitochondrii” cells (predators) and the number of mitochondria (prey) are linearly correlated. This result is not consistent with an oscillatory behavior of the “*Ca*. Midichloria mitochondrii”-mitochondrion system. Analyzing an oscillatory equilibrium of the Lotka-Volterra model, we found that it is unlikely to obtain a significant linear correlation between prey and predator by chance. This result suggests that the “*Ca*. Midichloria mitochondrii”-mitochondrion system within *I. ricinus* oocytes at previtellogenic and late-previtellogenic developmental stages is likely in a steady-state equilibrium: the total amounts of “*Ca*. Midichloria mitochondrii” and mitochondria are stable over time.

Recently, Summers and Kreft (16) proposed a mathematical model for the *Bdellovibrio* life cycle that includes the predator (*Bdellovibrio*), the prey, and the prey invaded by *Bdellovibrio* (forming the bdelloplast). They showed that a steady-state equilibrium for the system is rare, but possible. They described two possible steady-state equilibria: one characterized by a bigger amount of prey than *Bdellovibrio* and one by more *Bdellovibrio* than prey (16). In both the cases, the amount of bdelloplasts (prey invaded by *Bdellovibrio*) shows an intermediate value between those for *Bdellovibrio* and prey. As shown in Fig. S4, the number of colonized mitochondria (i.e., equivalent to the bdelloplast in our model) is lower than those of “*Ca*. Midichloria mitochondrii” (i.e., *Bdellovibrio* in the model) and mitochondria (i.e., the prey in the model), for all of the oocytes but one.

All these results show that our TEM data cannot be considered coherent with a *Bdellovibrio*-like behavior.

Finally, we would like to make a general consideration on the likelihood of a *Bdellovibrio*-like behavior in this biological system. An oocyte can be considered an island, where space is limited. Often it has been observed that in ecological situations of limited space and resources, predators are absent (17), possibly because it is not possible to maintain a stable equilibrium between prey and predator populations in such systems.

After rejecting the *Bdellovibrio*-like model, here we use the generated data to propose a novel model for the “*Ca*. Midichloria mitochondrii” life cycle within the *I. ricinus* oocytes.

### The “mitochondrion-to-mitochondrion” hypothesis.

Here, we propose a different hypothesis for the “*Ca*. Midichloria mitochondrii” life cycle within *I. ricinus* oocytes, developed on the basis of the collected data.

TEM evidence shows that a single mitochondrion can be colonized by up to 10 bacterial cells. In parallel, a small-sized bacterium (comparable to a mitochondrion) preyed on by B. bacteriovorus can bear only as many as three or four predatory bacteria before undergoing lysis (18). What could be the cause of such a great difference? One of the many differences between free-living bacteria (preys of *Bdellovibrio*) and mitochondria is that while a bacterium is an individual unit, spatially separated from the others, mitochondria often are not (13, 14). Indeed, the continuous process of mitochondrial fusion and fission leads to the generation of a mitochondrial network, in which mitochondria are connected (19). It is possible that when we observe several bacterial cells within a mitochondrion, the physical pressure due to the presence of these bacteria does not affect the membranes of the single mitochondrion but is distributed among those of the mitochondrial network.

### Could “*Ca*. Midichloria mitochondrii” pass from one mitochondrion to another?

We tested this hypothesis using stochastic simulations. The structure of mitochondrial networks can be highly dynamic and variable, but they have been modeled to follow well-known network topologies, such as the scale free and small world (19, 20). Thus, we decided to perform our simulations using three of the most studied network topologies: scale free, small world, and random (see Video S1 for a representation of different network topologies and movement of *Ca.* Midichloria mitochondrii).

The scale-free network simulation showed that the number of mitochondria colonized by at least one, two, three, etc., “*Ca*. Midichloria mitochondrii” cells decreases following a power law, fitting the data obtained through TEM observations. This result suggests that the mitochondrion-to-mitochondrion model can explain the distribution of “*Ca*. Midichloria mitochondrii” within mitochondria.

The simulations we performed in this work had the aim to test whether the TEM observations can be explained in accordance with the mitochondrion-to-mitochondrion model. For sake of simplicity, we performed the simulations without considering cytoplasmic “*Ca*. Midichloria mitochondrii” replication and mitochondrial dynamics. Thus, these simulations represent a rough description of the mitochondrion-to-mitochondrion model, and they cannot be considered an exhaustive description of the system.

The large majority of colonized mitochondria bear only one or few “*Ca*. Midichloria mitochondrii” cells and display a morphology comparable to that of noncolonized mitochondria. On the other side, the very few highly colonized mitochondria show degraded morphology (Fig. 1). This result is not surprising, as it is coherent with what Sacchi and colleagues (9) observed. This observation can be explained in two ways: (i) “*Ca*. Midichloria mitochondrii” preferentially invades mitochondria that are in an independent process of degradation or (ii) the presence of high numbers of “*Ca*. Midichloria mitochondrii” cells within a single mitochondrion can lead to organelle degradation. Unfortunately, at the state of the art, it is not possible to directly observe “*Ca*. Midichloria mitochondrii” life cycle within the tick oocyte; thus, it is not possible to test these hypotheses.

The intramitochondrial tropism of “*Ca*. Midichloria mitochondrii” makes this bacterium a model particularly interesting for the study of mitochondrial metabolism and physiology, not only in ticks but in general. Our work lays the foundation for quantitative approaches to investigate this system. In the future, studies to characterize mitochondrial dynamics in *I. ricinus* oocytes and more exhaustive mathematical models will be necessary to further elucidate this unique system.

## MATERIALS AND METHODS

### Electron microscopy experiments.

A total of 11 wild ticks of the species Ixodes ricinus, collected from Italy, France, Switzerland, and Czech Republic between 2002 and 2017 (see Table S2 for details, available at https://github.com/FrancescoComandatore/M.mitochondrii_TEM_count/blob/main/TableS2.xls), were included in the study. All of these ticks were subjected to ovary extraction, and the extracted material was included in EMbed 812 (epoxy resin) and subjected to transmission electron microscopy (TEM) observation using a Zeiss EM 900 microscope. In more detail, samples were fixed in 0.1 M cacodylate buffer (pH 7.2) containing 2.5% glutaraldehyde for 2 h at 4°C and postfixed in 2% OsO_4_ in 0.1 M cacodylate buffer for 1.5 h at 4°C. Subsequently, the samples were washed, dehydrated through a progressive ethanol gradient, transferred to propylene oxide, and embedded in Epon 812. Thin sections (80 nm) were stained with saturated uranyl acetate followed by Reynolds lead citrate and examined with a Zeiss TEM 900 transmission electron microscope at 80 kV.

From each ovary, intact oocyte sections were examined, meaning oocytes for which it was possible to clearly detect the entire cellular border. Among intact oocyte sections, only those at previtellogenic (P) and late-previtellogenic (LP) developmental stages were selected for the counting step described below. Eggs at previtellogenic (P) and late-previtellogenic (LP) stages were distinguished on the basis of the cytoplasm composition: in P cells, the cytoplasm contains amorphous areas and it is less rich in vitellogenin, while LP cells contain large vitellogenin globules. We focused on these two stages because they are the only ones in which “*Ca*. Midichloria mitochondrii” bacteria and mitochondria can be easily identified. Indeed, oocytes at earlier stages are too small to allow this kind of observation, while the cytoplasm of those at later stages is too rich in vitellogenin, which produces black electron-dense granules. For each selected oocyte section, the number of “*Ca*. Midichloria mitochondrii” bacteria observed outside mitochondria, the number of noncolonized mitochondria, and the number of mitochondria colonized by one, two, three, four, or five or more bacteria were counted. TEM does not provide direct evidence of mitochondrial functionality; however, to exclude as much as possible dead/senescent mitochondria, only those presenting intact membranes were counted.

### Real-time PCR assays.

Two semiengorged *I. ricinus* ticks were collected in northern Italy and manually dissected in order to retrieve, from each, 5 groups of ∼10 oocytes at previtellogenic/late-previtellogenic development stages, as it is not possible to distinguish vitellogenic states (i.e., P from LP oocytes) using optic/light microscopy.

Each group of oocytes was processed for DNA extraction using a proteinase K incubation protocol (final concentration, 10 ng/μl). Samples were subjected to Sybr green quantitative real-time PCR assay for *cal* (calreticulin gene of *I. ricinus*) and *COII* (mitochondrial cytochrome oxidase II of *I. ricinus*) according to the protocol (including primers) described previously by Sassera and colleagues (5). Serially diluted plasmid standards were used to set up the standard curve to obtain the efficiency and the dynamic range of the reaction. These two reactions presented comparable efficiencies and dynamic ranges (107% for *COII* and 107.5% for *cal*); thus, the absolute quantification of mitochondria was estimated as the ratio of *COII* to *cal*.

### Testing the presence of *Coxiella* spp. and *Rickettsia* spp. in Ixodes ricinus oocytes.

*I. ricinus* females have ∼100% prevalence of “*Ca*. Midichloria mitochondrii” in the ovary (4), but other bacterial species can colonize the tick ovary, in particular, *Coxiella* spp./*Coxiella*-like and *Rickettsia* spp./*Rickettsia*-like bacteria (1). We tested by PCR the presence of *Coxiella* spp./*Coxiella*-like and *Rickettsia* spp./*Rickettsia*-like bacteria in the 10 samples previously subjected to real-time PCR to quantify “*Ca*. Midichloria mitochondrii.” An ∼350-bp fragment of the citrate synthase gene (*gltA*) was amplified in order to detect the presence of *Rickettsia* spp./*Rickettsia*-like bacteria (21). A 524-bp fragment of the 16S rRNA gene was amplified to detect the presence of *Coxiella* spp./*Coxiella*-like bacteria (22).

### Development stage comparisons.

Data obtained from TEM experiments were used to count “*Ca*. Midichloria mitochondrii” cells and mitochondria within the sections of oocytes at the P and LP stages. In particular, the total numbers of “*Ca*. Midichloria mitochondrii” cells and mitochondria per oocyte section, the average total number of “*Ca*. Midichloria mitochondrii” cells, the average total number of mitochondria, the average number and frequency of intramitochondrial “*Ca*. Midichloria mitochondrii,” and the average number and frequency of colonized mitochondria were compared using the Wilcoxon test. This TEM-based counting approach has an intrinsic limit: it is based on two-dimensional images and does not allow observation of the entire mitochondrion. Considering that “*Ca*. Midichloria mitochondrii” and mitochondria are similarly sized, we can suppose that TEM observations provide a good estimation of the number of mitochondria colonized by at least one “*Ca*. Midichloria mitochondrii.” On the other hand, the observed number of bacteria present within a mitochondrion would represent the lower limit of symbionts actually present within the organelle. To overcome this limitation, the comparisons were performed considering the number and frequencies of mitochondria colonized by at least the number of counted bacteria.

### Analysis of the distribution of mitochondrial colonization levels.

Any mitochondrion within a single oocyte can be colonized by a number of “*Ca*. Midichloria mitochondrii,” which ranges from 0 to N (we will refer to this number as the mitochondrial colonization level [MCL]). To study the distribution of MCL in each oocyte, considering the intrinsic limit of TEM explained above, the complementary cumulative distribution (CCD) was calculated as follows:

(2) F(x)=P(X ≥ x),

where F(*x*) is the complementary cumulative frequency of mitochondria colonized by at least *x* “*Ca*. Midichloria mitochondrii” cells.

Then, the F(*x*) of each oocyte with at least three different mitochondrial colonization levels (i.e., with at least three points in the CCD plot) was tested for power law fitting using the linear least-squares method to a linear model obtained by a log-log transformation of the power law model in order to estimate its parameters.

(3) $$\text{F(}x)=\text{c}\times x^{-\alpha },$$

(4) log[f(x)]=log(c) + (−α)×log(x),

where *x* indicates the MCL value.

Once the α and c parameters had been estimated for each of these oocytes, their average values were compared between oocytes at the P and LP development stages using the Wilcoxon test and the *t* test.

A residual analysis was conducted to confirm the accuracy and validity of the fitting, evaluating residual homoscedasticity, normality, and no autocorrelation. The confidence interval for each fitted α parameter was considered, comparing confidence intervals of α parameters calculated on CCD with different numbers of MCL (i.e., different number of points in the CCD plot).

Furthermore, the average complementary cumulative frequency distribution (average CCD) of the MCLs (from 0 to 5 or more) was obtained as the average of the CCD distributions calculated for each oocyte. The obtained CCD was then tested for the power law fitting (as described above), and the α parameter was estimated as described above.

Lastly, the average number of mitochondria colonized by at least X bacteria was inferred as follows:

(5) $$\text{N(X)}=\text{M}\times {\text{(X}}^{-\alpha }\text{),}$$

where N(X) is the number of mitochondria colonized by at least X “*Ca*. Midichloria mitochondrii” cells, and M is the average number of mitochondria per oocyte measured by quantitative real-time PCR assay (see above).

### Comparing the number of cytoplasmic “*Ca*. Midichloria mitochondrii,” mitochondria, and colonized mitochondria.

The number of cytoplasmic “*Ca*. Midichloria mitochondrii” cells and the number of noncolonized mitochondria among all of the oocyte sections were compared by scatterplot and linear regression analysis, using R. The number of cytoplasmic “*Ca*. Midichloria mitochondrii,” noncolonized mitochondria, and colonized mitochondria were plotted using R (http://www.R-project.org/).

### Linear regression of prey and predator amounts in an oscillatory equilibrium of the Lotka-Volterra model.

Within each oocyte, the “*Ca*. Midichloria mitochondrii” life cycle runs over time, but unfortunately, each TEM experiment allows us to capture a single moment only. Despite this, we can infer some information about the behavior of the “*Ca*. Midichloria mitochondrii”-mitochondrion system using the collected data. For parsimony, we can presume that “*Ca*. Midichloria mitochondrii” has the same life cycle in all the *I. ricinus* oocytes and each TEM experiment stopped the oocyte in a random step of this cycle. The observation of several randomly distributed steps of the cycle can be used to infer some properties of the “*Ca*. Midichloria mitochondrii”-mitochondrion system. Here, we used the collected data to infer if the system has an oscillatory behavior, even if the counted “*Ca*. Midichloria mitochondrii” cells are linearly correlated with the counted mitochondria. Sacchi and colleagues (9) hypothesized the “*Ca*. Midichloria mitochondrii” could have a predatory-like behavior, invading and consuming mitochondria. We studied this hypothesis using an oscillatory prey-predator Lotka-Volterra model. We computed the probability that, collecting a certain number of time points from an oscillatory prey-predator time series, the collected prey and predator numbers are linearly correlated.

More in detail, we evaluated the probability to observe a significant linear regression between the number of “*Ca*. Midichloria mitochondrii” cells and mitochondria from *N* randomly sampled time points of a system in oscillatory equilibrium, considering the following *N* values: 10, 20, 30, 40, 50, 60, 70, 80, 90, 100, 110, 120, 130, 140, and 150. We considered an oscillatory equilibrium of the prey-predator Lotka-Volterra model:

(6) $$\frac{\mathrm{dx}}{\mathrm{dt}}=\alpha \times \beta -\beta \times x\times y,$$

(7) $$\frac{\mathrm{dy}}{\mathrm{dt}}=\gamma \times x\times y\;-\;\delta \times y.$$

Setting the parameters of α to 2, β to 0.7, γ to 0.5, and δ to 0.8 for 500 time steps. We randomly sampled the time points among the 500 time steps, for 10,000 bootstrap replicates. For each replicate, we computed the *P* value of the linear regression between prey and predator amounts. Then, we computed the frequency of the 10,000 bootstrap replicates for which the linear regression analyses returned a *P* value of <0.05, <0.01, or <0.001. The analyses were computed using R.

### Regression analysis of the frequency of intramitochondrial “*Ca*. Midichloria mitochondrii” and colonized mitochondria.

The frequencies of intramitochondrial “*Ca*. Midichloria mitochondrii” and the frequency of colonized mitochondria among all of the oocytes were compared by using scatterplot and linear regression analysis, both performed using R.

### Mathematical simulation of the spread of “*Ca*. Midichloria mitochondrii” through mitochondrial networks.

Mitochondrial networks can be studied using graphs and mathematical structures composed of nodes connected by edges (23). Despite the fact that mitochondrial networks are highly dynamic, for simplicity, the movement of “*Ca*. Midichloria mitochondrii” within the host mitochondrial network was simulated using a static graph. Without *a priori* knowledge about mitochondrial dynamics in tick oocytes, we decided to perform three independent simulations using mitochondrial graphs with three of the most studied topologies: scale free, small world, and random.

The number of nodes (N) and intramitochondrial bacteria (K) of each graph were estimated on the basis of real-time PCR data and TEM data collected as described above. In particular, N was estimated on the basis of the average *COII*/*cal* value measured by real-time PCR experiments (see above), and the K parameter was estimated as follows:

(8) $$\text{K}=\text{N}\times \left\{\sum_{u=1}^{\text{W}}\left[\left(\sum_{i=1}^{5}{\text{M}}_{u,i}\times I\right)/\sum_{i=1}^{5}{\text{M}}_{u,i}\right]\right\}/\text{W,}$$

where W is the number of oocytes included in the study, and M is the number of mitochondria within the oocyte *u* colonized by *i* bacteria.

For each simulation, the following algorithm was used:

1. A graph of N (previously estimated) nodes was generated using R; the nodes represent the mitochondria, and the edges represent the connections among them.
2. K (previously estimated) nodes of the graph were randomly selected to harbor one “*Ca*. Midichloria mitochondrii.”
3. Each “*Ca*. Midichloria mitochondrii” was allowed to move from its node to a randomly selected neighbor node on the graph, with a probability of 0.1.
4. Step 3 was repeated 1,000 times.
5. The frequency of each MCL (from 0 to N) was calculated for the mitochondrial population at the last step.

Steps 1 to 5 were repeated 10,000 times.

Scale-free networks were generated using the barabasi.game function (parameters: M, out.pref, FALSE; power, 0.8; zero.appeal, 2; m, 3), small-world networks were generated using the function watts.strogatz.game (parameters: dim, 1; size, M; nei, 5; *P* = 0.05), and random networks were generated with the erdos.renyi.game function (parameter: M, p.or.m = 0.5), all from the R package igraph (24). For each of the three simulations, and for each step of the simulation, the frequency CCD of the MCL was obtained for each network, and then the average frequency CCD was calculated. Furthermore, the average frequency CCDs obtained at the last step were compared to the average frequency CCD obtained from TEM experiments (see above), using Spearman’s rank correlation test.

10.1128/mBio.00574-21.8 VIDEO S1 Simulated mitochondrial networks and mitochondrion-to-mitochondrion movements of “*Ca*. Midichloria mitochondrii” cells. The video shows the movement of a population of 48 “*Ca*. Midichloria mitochondrii” cells on three mitochondrial networks characterized by different topologies: scale free on the top, random on the left, and small world on the right. Each node of the network is a mitochondrion, and uncolonized mitochondria are colored black. Nodes colonized by “*Ca*. Midichloria mitochondrii” are colored on the basis of the network topology: red for scale free, green for random, and brown for small world. The node size is proportional to the number of colonizing “*Ca*. Midichloria mitochondrii” cells. Download Movie S1, MOV file, 11.0 MB. Copyright © 2021 Comandatore et al. 2021 Comandatore et al. https://creativecommons.org/licenses/by/4.0/ This content is distributed under the terms of the Creative Commons Attribution 4.0 International license.
