# Supplementary figures and images for: Modeling the Life Cycle of the Intramitochondrial Bacterium “Candidatus Midichloria mitochondrii” Using Electron Microscopy Data
Source: mBio. 2021 Jun 22;12(3):e00574-21. doi: 10.1128/mBio.00574-21 (PMC8262999; doi:10.1128/mBio.00574-21)

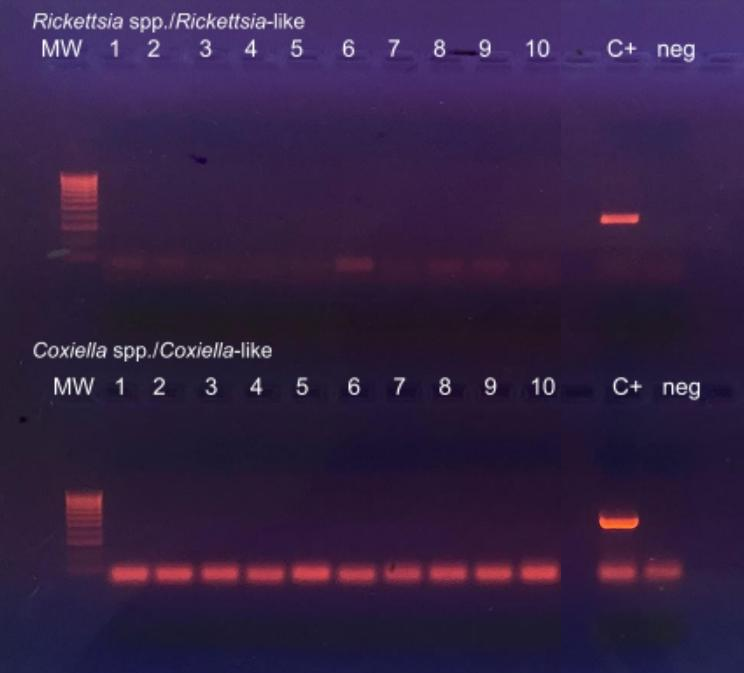

Supplement: FIG S1 [file mbio.00574-21-sf001.tif]

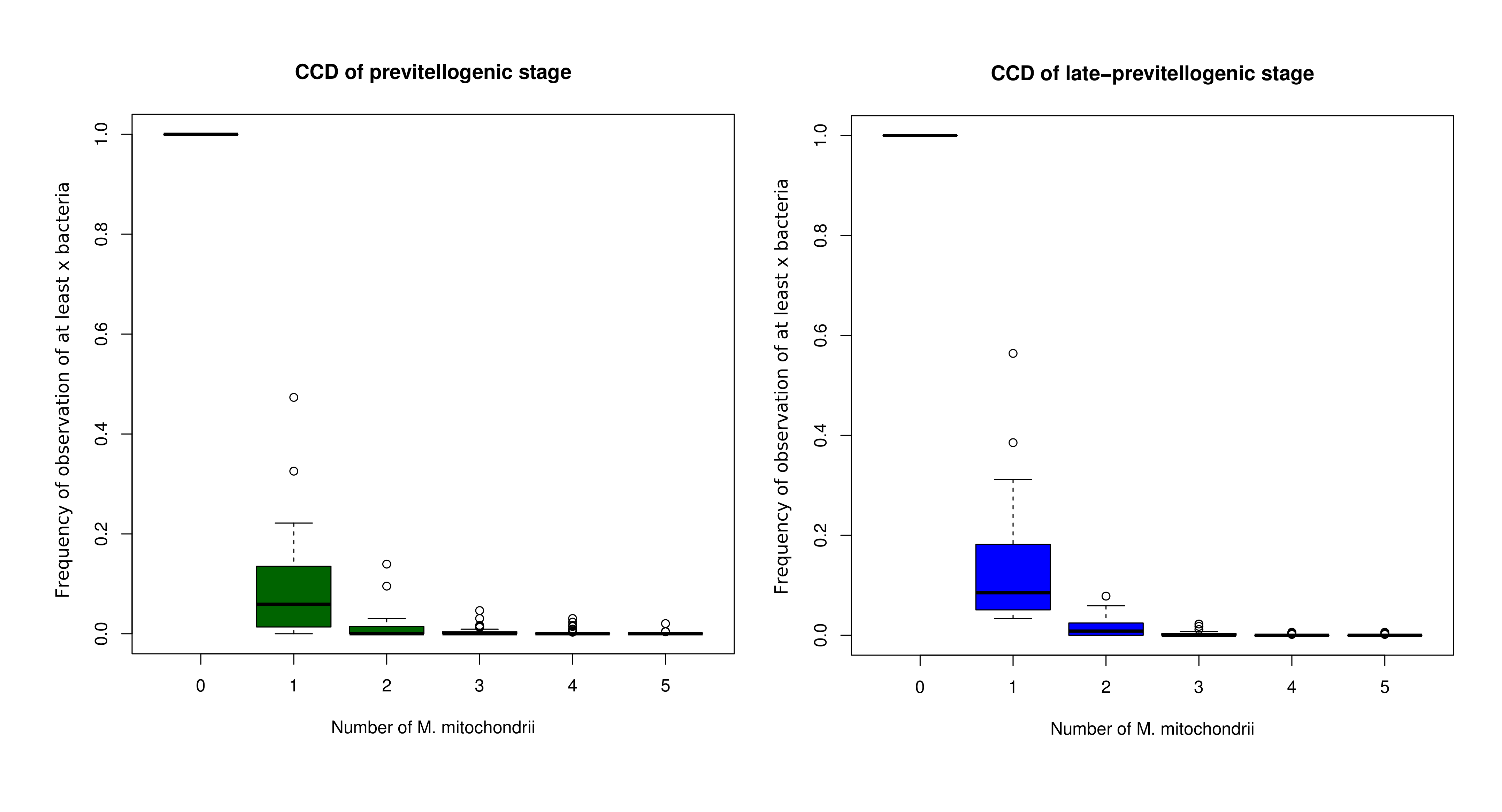

Supplement: FIG S2 [file mbio.00574-21-sf002.tif]

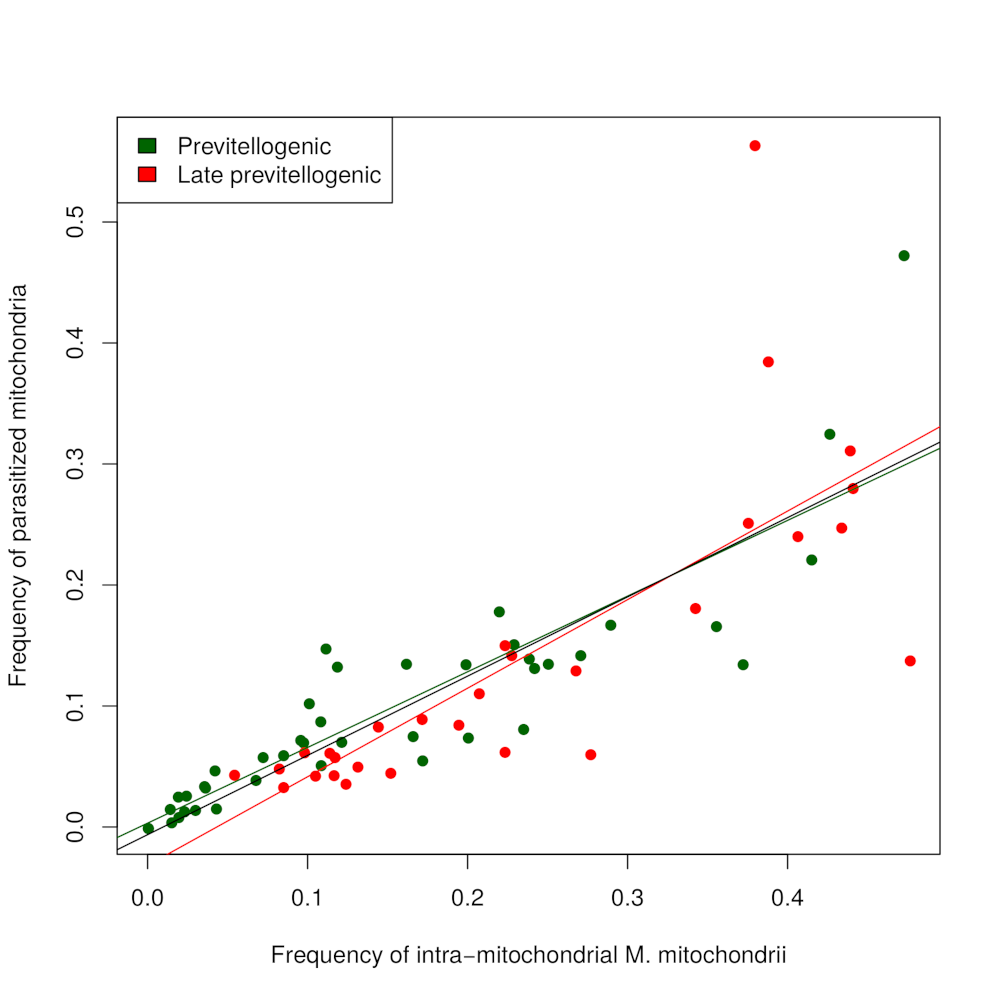

Supplement: FIG S3 [file mbio.00574-21-sf003.tif]

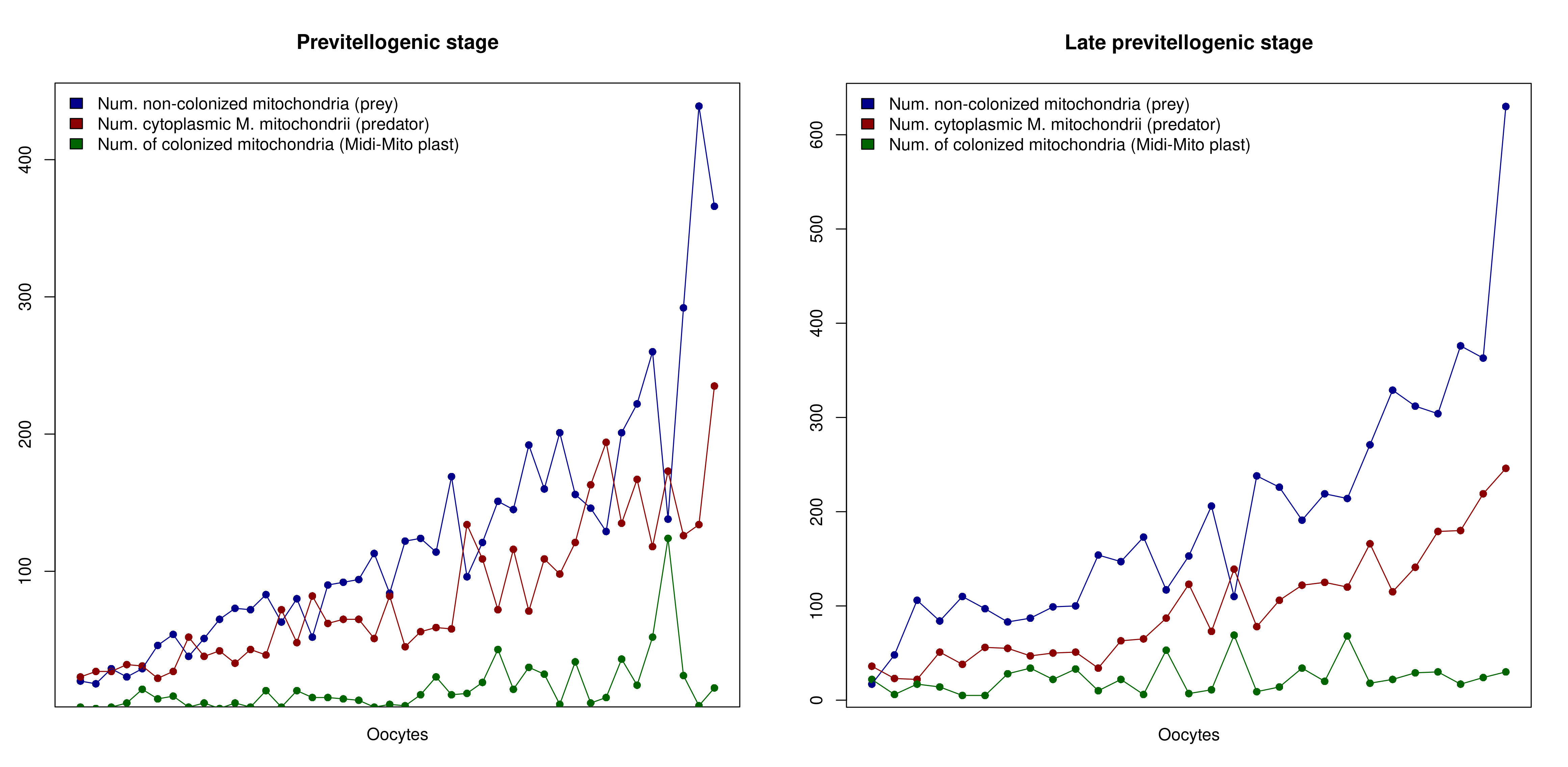

Supplement: FIG S4 [file mbio.00574-21-sf004.tif]

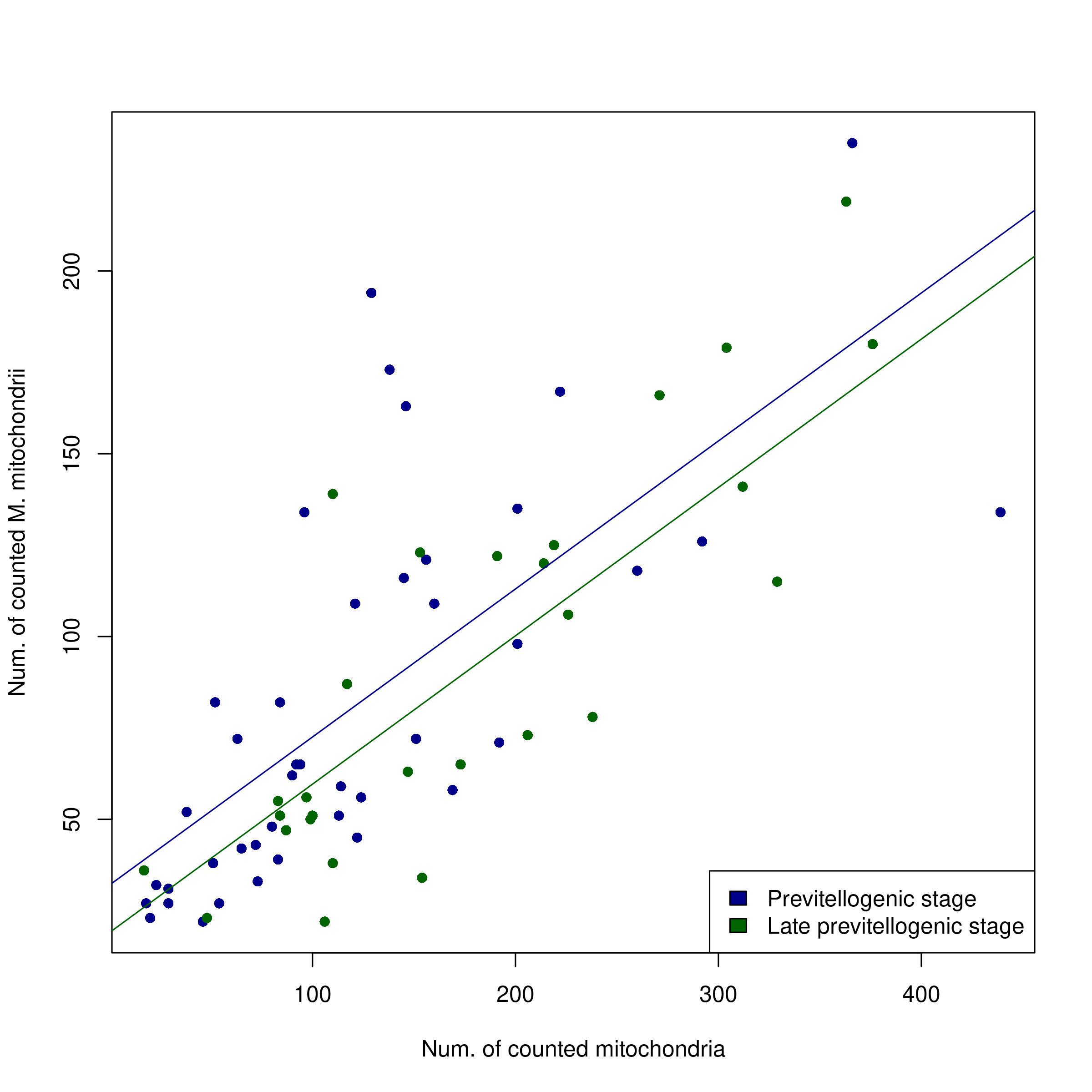

Supplement: FIG S5 [file mbio.00574-21-sf005.tif]

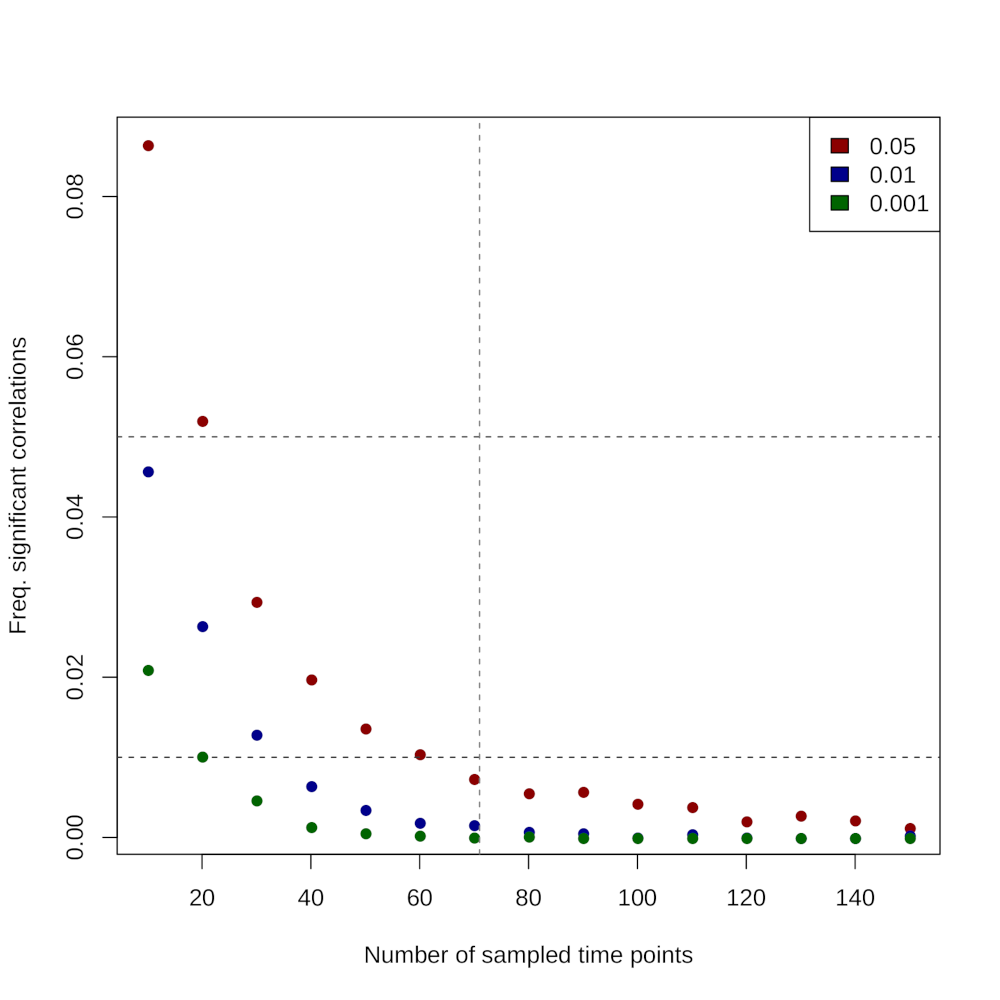

Supplement: FIG S6 [file mbio.00574-21-sf006.tif]

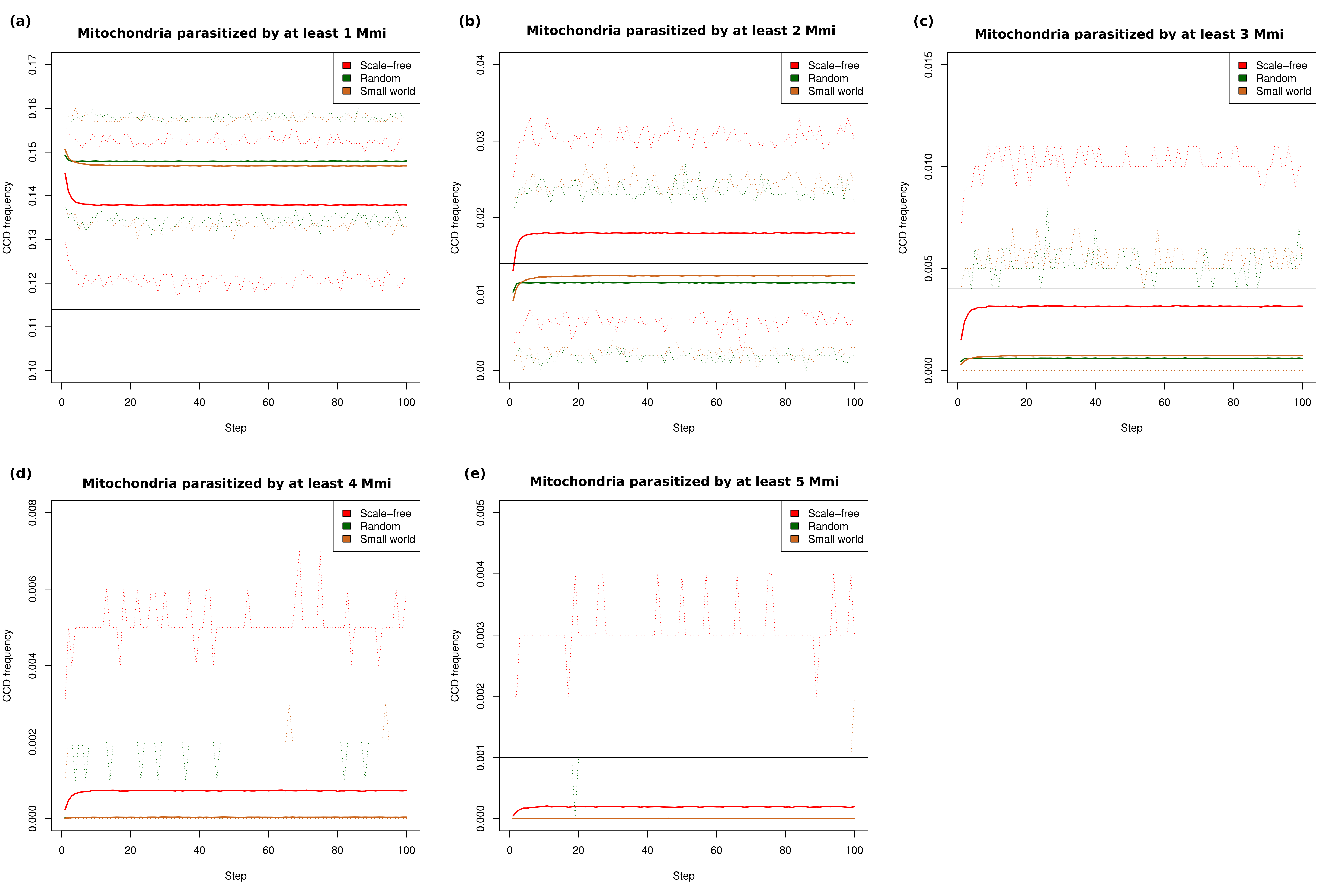

Supplement: FIG S7 [file mbio.00574-21-sf007.tif]
